# Simulation studies on the boot shape injection of a giant magnetostrictive injector

**DOI:** 10.1038/s41598-021-02529-z

**Published:** 2021-11-26

**Authors:** Guangming Xue, Jitao Ge, Peng Ning, Jun Zhou, Ke Wang, Ziyang Cheng, Guoxu Pei

**Affiliations:** 1 People’s Liberation Army Troop 63969, Nanjing, 210028 China; 2 Sergeant School, University of Aerospace Engineering, Beijing, 102249 China

**Keywords:** Electrical and electronic engineering, Mechanical engineering, Software

## Abstract

Giant magnetostrictive injector using giant magnetostrictive material acting an electronic controlled injector may be one new promising injector to acquire adjustable injection rates while maintaining large injection quantity. An electronic controlled injector driven by a giant magnetostrictive actuator was designed through combining the driving requirement and output characteristics of the material. To promote responding speed of the coil current, the driving voltage with open-hold-fall type waveform was employed just like using in an electromagnetic injector. Simulation model for the injection characteristic of the injector was established using AMEsim software and verified using experimental results collected by the single injection meter. From simulation and experimental results, designed giant magnetostrictive injector showed good performances as maximum spray rate of 4.5 L/min and minimum spray pulse width of 0.21 ms, and realized the boot shape injection when generated by the designed voltage wave. Furthermore, duration time and amplitude of the pilot spray part in a boot shape injection were respectively adjusted through changing the dwell time and opening time. The boot shape injection reached by the giant magnetostrictive injector can reach quite accurate control of fuel injection and then promote fuel efficiency effectively.

## Introduction

The global problems of fossil energy resource reduction and air contamination call for cleaner energy vehicles with less fuel consumption and lower exhaust gas emission. In recent years, formulated stricter vehicle emission standards and rapid development of the electrically driven vehicle have set higher requirements for the fuel ignition vehicles^1,2^. Further reducing the fuel consumption and pollutant emission has been the only opportunity for the fuel ignition engines to struggle for its place from other types of clean energy vehicles. Exploring new fuels and improving the fuel injection system are two important approaches to reach a more environmentally friendly fuel ignition engine. Giant magnetostrictive injector (GMI) which employs the giant magnetostrictive material as its core component may overcome the problems occurring in an electromagnetic injector (slow response and low controllability) or a piezoelectric injector (require a hydraulic amplifier and lose efficacy easily), and be one promising candidate of the electronic controlled injector.

Giant magnetostrictive material is a kind of energy material and has some advantages as high coupling coefficient, fast response speed, huge output force and high Curie temperature etc.^3,4^. This kind of material has been widely used in active vibration control, driving hydraulic devices, ultra precision machining and some other areas^5–11^. Compared to the electromagnetic injector, giant magnetostrictive type of injector can be regarded as an advanced electromagnetic injector with continuously adjustable displacement in the drive part. In addition to maintaining the advantages of large injection rate, high working stability and strong anti-interference ability, giant magnetostrictive type of injector can reach more flexible injection rate and has the feasibility of direct acting structure.

Giant magnetostrictive type of injector has been noticed by many researchers. Li et al., Wang et al., Lv et al., Pîslaru-Dănescu L et al.^12–17^ respectively proposed their designs of the actuator used on the electronic controlled injector and gave some useful simulation and experimental results of magnetic fields or displacements. Bright et al.^18^ stated that giant magnetostrictive material was helpful for better combustion control and gave their structure design of programmable fuel injector. In addition, they supplied an effective driving voltage profile for the GMI. Yan et al.^19^ designed a giant magnetostrictive fuel injector and used finite element method analyzing its magnetic field. Tanaka et al.^20,21^ developed a common-rail proportional injector driven by tandem arrayed giant magnetostrictive actuator, which can reach the displacement of maximum value of 50 μm using six giant magnetostrictive rods with length of 30 mm. They also established the bond-graph model of designed injector and supplied some experimental results of the transient-state process. He et al.^22–26^ presented some useful information on the aspects of structure design, driving voltage optimization and some computational methods on the giant magnetostrictive actuator used for the injector. Xu et al.^27^ proposed the new GMI with a flexible reversing amplifying mechanism, which promoted the maximum displacement of the giant magnetostrictive actuator from 0.12 mm to 0.48 mm from their COMSOL simulation model. Besides these researches, plenty of patents of the GMI were also presented, which could provide many useful structure design ideas^28–32^.

Above literatures mainly studied the design, modeling and simulation of the whole GMI or some testing of the giant magnetostrictive actuator, while involved less experimental verifications. Meanwhile, spray potential of the GMI was not exploited that this new type of injector was just acting like an electromagnetic injector.

This paper presented a novel indirect acting GMI, which made use of a zero biased giant magnetostrictive actuator. To promote the responding speed of the coil current, the open-hold-fall type driving voltage was employed to stimulate the injector. The AMEsim simulation model for the whole injector was established and verified with help of a high pressure common rail experimental system. Then spray characteristics of the GMI when executing the boot shape injection was analyzed using the AMEsim model. And adjustable height and length of the pilot spray part were reached by designed magnetostrictive injector from simulation. For the GMI, this paper can provide some new ideas of the injector structure and driving voltage waveform, which can effectively reach new types of spray methods for future higher fuel efficiency.

## Structure and driving voltage of the GMI

### Structure design

**Figure 1:**
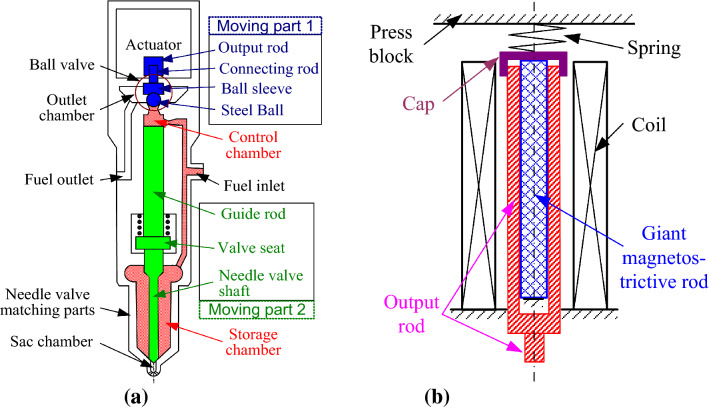
Structure profile of the GMI: (**a**) structure drawing of the whole injector; (**b**) structure drawing of the actuator part.

The structure profile of proposed GMI was shown in Fig. 1. There are two moving assemblies respectively of part 1 including output rod, connecting rod, ball sleeve and steel ball, and part 2 including guide rod, valve seat and needle valve shaft. The control chamber and storage chamber should be focused to understand the operating principle of the GMI easily shown in Fig. 1a.

- When the giant magnetostrictive actuator was electrified with the power on, the ball valve was opened and the fuels in the control chamber relieved its pressure. Then the upward forces exerted on the part 2 were higher than downward ones that the moving part 2 would be pushed upward and the injector began spraying fuels.
- With the power off, the actuator pushed the steel ball downward to close the ball valve. Then the fuel in control chamber accumulated the pressure gradually until its pressure equal to that in storage chamber. In this process, the downward forces exerted on moving part 2 were higher than upward ones and pushing the moving part 2 downward to close the needle valve. Then the injector stopped spraying.

The actuator was the electronic controlled core to open the ball valve with power on and to close the ball valve with power off. Fig. 1b demonstrated the working principle of the giant magnetostrictive actuator. The lower end of the giant magnetostrictive rod was fixed to the still block and the upper end pressed on the cap which was in threaded connection to the output rod. With the coil electrified, the unbiased giant magnetostrictive rod overcame the pre-tightening force of the spring and elongated its length. Then the output rod together with the cap was pushed upward. When power was off, the GMM rod shortened and the output rod was moved to initial position by the spring.

### Driving voltage

**Figure 2:**
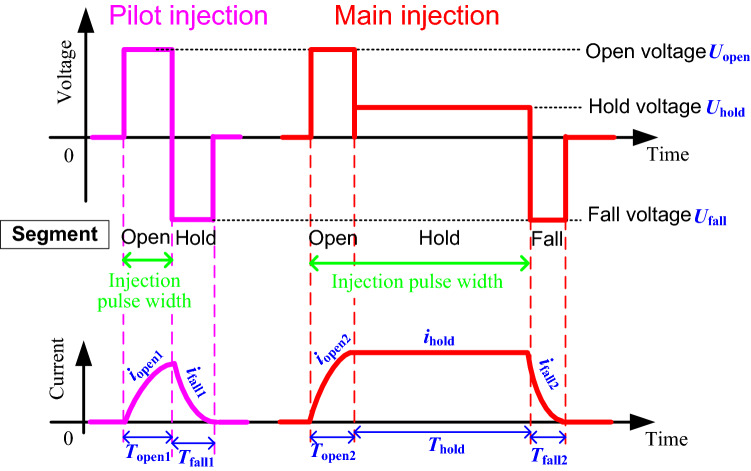
Voltage waves for a pilot injection and a main injection, respectively.

High inductance coil utilized in the GMI increased the responding time of the actuator to an unacceptable level. To promote the responding speed of the injector, the open-hold type voltage commonly used in an electromagnetic injector was introduced^23,24^. Waveforms of the open-fall type and open-hold-fall type voltages suitable to the GMI were designed and shown in Fig. 2. The open-hold-fall type voltage was used to execute a main injection. The injections with different pulse widths could be acquired through adjusting the hold time. Removing hold segment, the open-fall type voltage was used to execute a pilot injection or other type of the short injection like a post injection. Compared with the driving waveform of the voltage used in an electromagnetic injector, optimal driving voltage for the GMI removed its dwell time between the open and hold segments.

The parameters of the voltage waveform are calculated as follow. For the open-hold-fall type voltage, the open duration *T*_open2_ is better raising the coil current (or the displacement) just right from 0 to its steady state value, as more adjusting time will be needed for the coil current to reach steady state value when the duration is higher or lower. Firstly, the coil current *i*_open2_ in the open segment can be calculated from

1 $$L\frac{\mathrm{d}{i}_{open2}}{\mathrm{d}t}+R{i}_{open2}={U}_{open}$$

where *L* and *R* represent the coil inductance and resistance, *U*_*open*2_ is the open voltage. Consider initial value of the current is 0, one gets

2 $${i}_{open2}=\frac{{U}_{open}}{R}\left(1-{e}^{-\frac{R}{L}t}\right)$$

Impose Eq. (2) to be the steady-state current in the value of *U*_*hold*_/*R*, where *U*_*hold*_ is the hold voltage, the most reasonable open duration *T*_*open*2_ can be solved.

In the hold segment, the coil current remains the value of *U*_*hold*_/*R* with the duration of *T*_*hold*_, which denotes the duration of the hold voltage. Then the fall current will inherit the hold current as its initial value, and the fall current can be calculated as

3 $${i}_{fall2}=-\frac{{U}_{fall}}{R}+\frac{{U}_{fall}+{U}_{fall}}{R}{e}^{-\frac{R}{L}t}$$

Similarly, the fall voltage with the amplitude of *U*_fall_ is better reducing the steady state current to 0 just after the duration *T*_*fall*_.

For the pilot injection, the open current has the same expression as Eq. (2) while the duration time *T*_*open*1_ is not restricted. Then the initial value of fall current will be the last current value in open segment as $\frac{{U}_{open}}{R}\left(1-{e}^{-\frac{R}{L}{t}_{open1}}\right)$. The expression of fall current *i*_*fall*1_ is solved as

4 $${i}_{fall1}=-\frac{{U}_{fall}}{R}+\left[\frac{{U}_{open}}{R}\left(1-{e}^{-\frac{R}{L}{t}_{open1}}\right)+\frac{{U}_{fall}}{R}\right]{e}^{-\frac{R}{L}t}$$

Impose Eqs. (3) and (4) as 0 respectively, the reasonable fall duration *T*_fall1_ in a pilot injection and *T*_fall2_ in a main injection were acquired. Combined with reasonable open duration *T*_*open*2_ solved from Eq. (2), one gets

5 $$\left\{\begin{array}{l}{T}_{fall1}=\frac{R}{L}\mathrm{ln}\left[1+\frac{{U}_{hold}}{{U}_{open}}\left(1-{e}^{-\frac{R}{L}{t}_{open1}}\right)\right]\\ {T}_{fall2}=\frac{R}{L}\mathrm{ln}\left(1+\frac{{U}_{hold}}{{U}_{open}}\right)\\ {T}_{open2}=\frac{R}{L}\mathrm{ln}\left(1-\frac{{U}_{hold}}{{U}_{open}}\right)\end{array}\right.$$

Overall, to execute efficient main and pilot injections, the voltages in different segments and their durations are better meeting the requirements shown in Eq. (5).

## Simulation and test

### AMEsim simulation model

**Figure 3:**
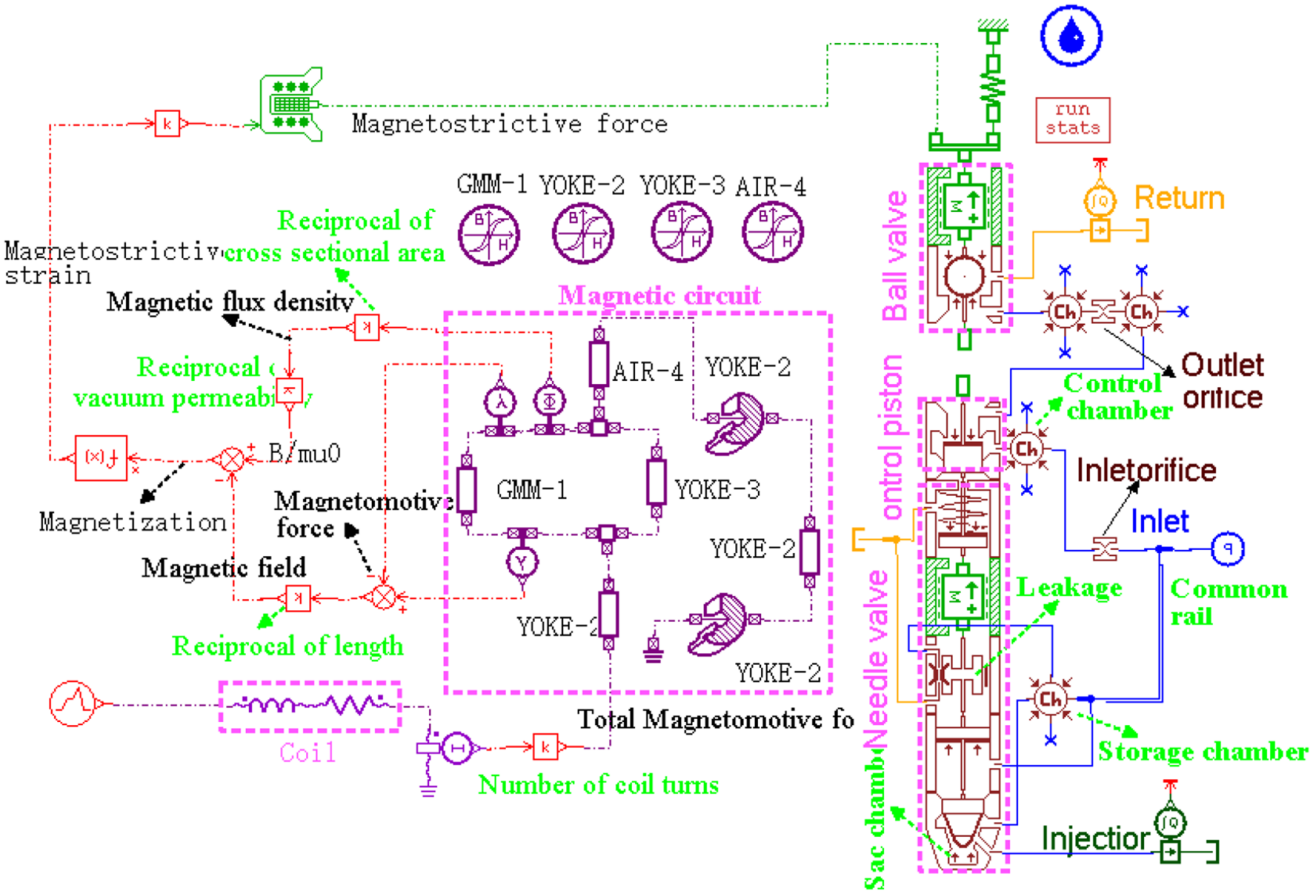
AMEsim model for the GMI.

AMEsim software is employed in this paper to calculate the spray characteristics of the GMI for its convenient and comprehensive simulation for a general mechanical–electrical-hydraulic system. The fully established injector simulation model is shown in Fig. 3.

In the AMEsim simulation, the coil is equivalent into series connection of a resistor and an inductor. Computations for the magnetic field and magnetization within the giant magnetostrictive material are based on the structure shown in Fig. 1b. The nonlinear magnetic characteristics including hysteresis of the material is considered through defining the material by the static Jiles–Atherton model. Then the sub-models of magnetostriction and force are established respectively using quadratic domain-rotation model and the assumption that the giant magnetostrictive rod is equivalent to a mass-spring-damper element. For the fuel passage part of the injector, the simulation model of the electromagnetic type injector embedded in AMEsim software is employed with necessary changes to match the real object.

Some important parameters of the ball valve in the simulation model are set as follows. For the GMI, the magnetostrictive force is connected to mass of the moving part 1. Just as Fig. 1a shows, the mass utilizes the mass sum of the output rod, connecting rod, ball sleeve and steel ball. No displacement limiting parts are employed in the actuator that the higher displacement limit of this mass should be set enough high. The lower limit remains 0 as the lower end of moving part 1 (the steel ball) is pushed against to the fixed seat. The return spring above the mass takes the value of the parallel stiffness of giant magnetostrictive rod and preload spring.

According to the situation of the subsequent experiments, the following conventions were followed during modeling.

- Giant magnetostrictive material was defined from static Jiles–Atherton model by The Magnetic Material Parameter Setting Tool in AMEsim. This definition considered the hysteresis loss while neglected the effect of the temperature on the output performance of the giant magnetostrictive material.
- The fuel pressure fluctuation of the common rail during single simulation and the influence of flow limiting valve on the injection characteristics of the injector were ignored.
- The model considered the long pipe pressure loss between the common rail pipe and the injector and the fuel leakage between the guide rod and the injector body. The pressure loss and leakage at other positions were also realized in simulation by the built-in function of AMESim element.
- AMESim's built-in Robert Bosch adiabatic diesel was directly used as the fuel model in the simulation, which ignored the heat exchange between the fuel and the external environment while considered the influence of fuel pressure on the flow state and added the influence of gas release and cavitation.

### Test setup

**Figure 4:**
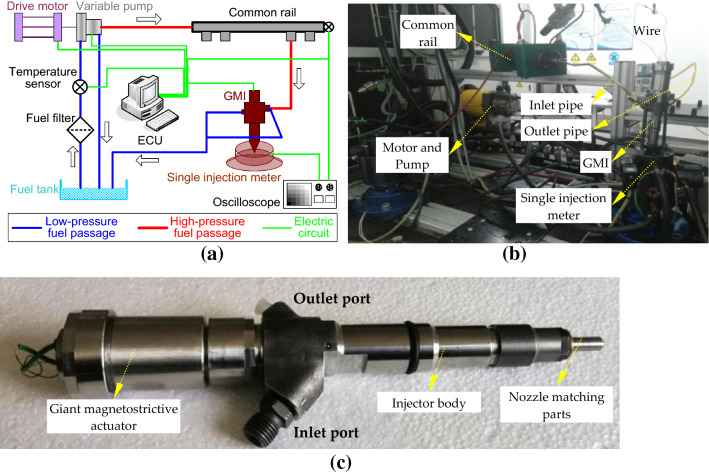
Experimental setup for the GMI: (**a**) schematic diagram of test system; (**b**) instrument hardware diagram; (**c**) GMI prototype.

To verify the simulation model, the spray characteristics of the GMI were tested with the help of the experimental setup (F-69390) of China North Engine Research Institute (Tianjin), just as shown in Fig. 4.

After the fuel filtration, the low-pressure fuel in the tank is accumulated by a variable pump and then stored in common rail. High-pressure fuel is connected with the inlet orifice of the injector through a long pipe. The return fuels from the pump and injector go through the low-pressure pipe to the fuel tank. The injection rate, quantity and duration time of the injector are collected using the single injection meter (EMI2) and the injection rate can be exhibited directly by an oscilloscope. The electronic controlled unit (ECU) is responsible for producing control signals for the drive motor and the injector.

The fuel injector body mainly adopted the body of Bosch's-212 type of high pressure common rail injector and the structural parameters in the simulation model were consistent with the object. The fuel used in the experiment was the calibration fluid supplied by China North Engine Research Institute (Tianjin). This type of fluid had similar physical properties to diesel and was also approximately consistent with the Robert Bosch adiabatic diesel selected in the simulation model. In addition, the duration of continuous test was effectively controlled to ensure that the monitored temperature was not too higher than 40 °C set in the simulation, and then the physical properties of the fuel or giant magnetostrictive material affected by the temperature in the test could be ignored. Overall, the experimental conditions were in good agreement with the simulation model shown in Fig. 3.

Main parameters of the GMI were tabulated in Table 1. One of the most important parameters was the dimension of the giant magnetostrictive rod as it determined the maximum elongation and force of the actuator. To reach maximum output displacement of 55 μm and maximum force not less than 30 N, the cylindrical giant magnetostrictive rod was chosen with length of 39.5 mm and diameter of 5 mm.

**Table 1:** Key parameters of the GMI.

Parameter (unit)	Value
**Giant magnetostrictive material**	
Type	Terfenol-D
Saturation magnetization (A/m)	8.0 × 10^5^
Shape parameter (A/m)	9800
Energy to break pinning sites(A/m)	1300
Reversibility coefficient	0.28
Domain interaction quantifier	− 0.001
Elastic modulus of material (N/m^2^)	3.0 × 10^10^
**Actuator part**	
Diameter of giant magnetostrictive rod (mm)	5
Length of giant magnetostrictive rod (mm)	39.5
Coil inductance (mH)	6.679
Coil resistance (Ω)	6.854
Mass of moving part 1 (g)	20
Stiffness of the spring in the actuator (N/m)	1.493 × 10^7^
Pre-tightening force of the spring in the actuator (N)	120
**Fuel passage part**	
Mass of moving part 2 (g)	35
Stiffness of needle valve spring (N/m)	5 × 10^4^
Pre-tightening force of needle valve spring (N)	50
Spray holes number × diameter (mm)	8 × 0.158
Flow coefficient into maximum cavitation for orifices of spray holes	0.75
Inlet orifice diameter (mm)	0.22
Maximum flow coefficient of inlet orifice	0.65
Outlet orifice diameter (mm)	0.28
Maximum flow coefficient of outlet orifice	0.65
Fuel temperature (fixed) (degC)	40.0

## Results and discussions

### Main and short sprays

**Figure 5:**
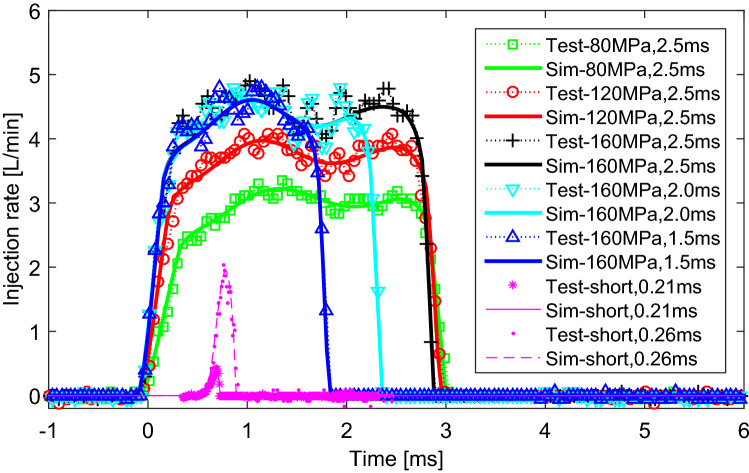
Injection rate profiles of test vs. that of simulation of main and short sprays.

Test on the GMI was done under main and pilot types of sprays by the voltage waves shown in Fig. 2. For the driving voltage, the amplitudes of the voltage in open, hold and fall segments are set as 80 V, 18 V and − 96 V respectively and duration times obey Eq. (5). Change common rail fuel pressure from 80 to 160 MPa spray pulse width from 1.5 to 2.5 ms respectively, injection rate profiles of the main sprays were drawn in Fig. 5. In addition, the pilot injection rate profiles with pulse width respectively of 0.21 ms and 0.26 ms were also plotted in the figure.

From the experimental and simulation results of main sprays with different fuel pressures, proposed GMI can reach stable injection rate from 3.0 to 4.5 L/min with the common rail pressure increased from 800 to 160 MPa approximately. Such high injection rate is higher than that of most market injectors, which demonstrates the excellent performance of high flow injection of designed injector. From the results of main sprays with different pulse widths, the spray duration time can be effectively adjusted by adjusting the hold time of the driving voltage.

From the experimental and simulation results of pilot sprays, the GMI can realize various triangular sprays using pilot injections as long as the pulse width is higher than 0.21 ms, which shows its fast responding speed. At the same time, the injector can control the fuel consumption quite accurately as the minimal single injection quantity is less than 10 mm^3^ (4.5 mm^3^ from experiment). Combined with the main injection results, the GMI can realize large flow and high-precision injection at the same time.

These results show good performances of designed GMI. Furthermore, from the comparisons between simulation and experimental results, the simulation model can predict the injection characteristic of the injector quite effectively as the curve shapes, spray durations and amplitudes of simulated injection rates are corresponded to experimental ones.

### Double and boot shape sprays

From above experimental and simulated results, designed GMI has fast response speed and high injection rate. There is no doubt that the injector can carry out a multi-injection easily. Besides the multi-injection, a boot shape injection can also be realized by the GMI. In a boot shape injection, the pilot injection is not completely parted from the main injection that there is no dwell time between the two injections. Increasing the duration of the pilot injection to a certain degree can change a double-injection into a boot shape injection.

**Figure 6:**
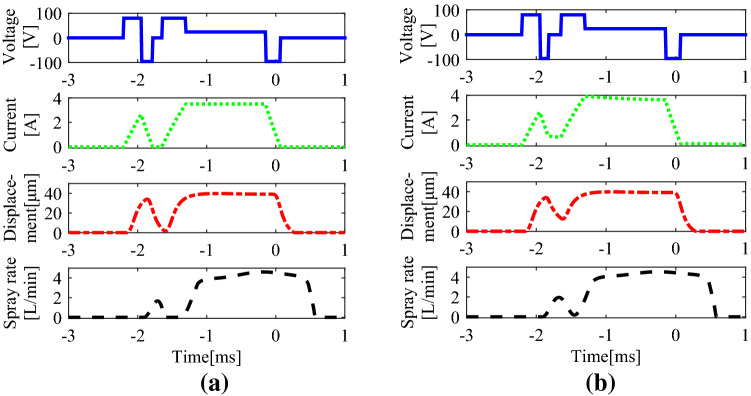
Simulation profiles of the voltage, coil current, ball valve lift and spray rate of the double injection and boot shape injection: (**a**) double injection; (**b**) boot shape injection.

GMI can execute controllable boot sprays as the output force or displacement of the high responding speed actuator is completely controllable in all the increasing or decreasing process. Set voltage amplitudes in open, hold and fall segments as 80 V, 18 V and − 96 V respectively and the duration time of different segments as shown in Table 2. For the double injection, time parameters were computed from Eq. (5). And the time parameters were determined from adjusting the fall time in the pilot segment and dwell time to make a fair comparison with the double injection. From simulation model, designed GMI executed the double injection and boot shape injection just as shown in Fig. 6.

**Table 2:** Simulation parameters for the double injection and boot shape injection.

Segments	Double injection	Boot shape injection
**Pilot segment (ms)**		
Open time	0.25	0.25
Fall time	0.168	0.12
**Dwell segment (ms)**		
Dwell time	0.132	0.18
Open time	0.348	0.348
**Main segment (ms)**		
Hold time	1.15	1.15
Fall time	0.217	0.217

Figure 6 supplied the curves of four important parameters respectively of the driving voltage, coil current of the actuator, ball valve lift and spray rate. From simulation results, reducing the fall duration in the pilot voltage decreases the fall speeds of the coil current and also of the ball valve lift. Then the pilot injection rate will not reduce to 0 when the main injection starts and the boot shape injection can be realized. More important, this kind of boot shape injection reached by designed magnetostrictive injector is completely controllable. It can be concluded directly that the spray rate of a boot shape injection can be adjusted as required through adjusting the driving voltage.

**Figure 7:**
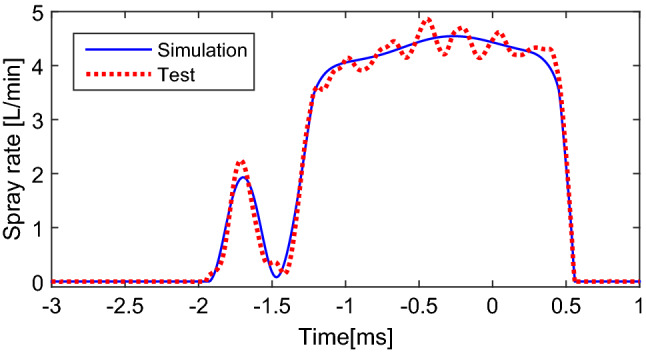
Injection rate profiles of test vs. that of simulation of boot shape injection under fixed parameters.

From Fig. 6, the driving voltage waveform used for the boot shape injection or double injection is actually a combination of the ones of the pilot injection and main injection. The control box generating driving voltage was developed with the help of HANDE Electric Appliance Co., Ltd. When employed for the boot shape injection, the control box generated the voltage wave with fixed parameters just as the shown in the last column of Table 2. The ECU controller shown in Fig. 4a was replaced with developed control box, and the common rail was always filled with high-pressure fuel. The spray rate profiles of the boot shape injection were drawn in Fig. 7.

From the experimental results shown in Fig. 7, the GMI executed the boot shape injection as design with stimulating by the voltage wave, which verified the feasibility of realizing a boot injection using this type of injector. Furthermore, as the simulation results were in good agreement with the experimental results, the effectiveness of the simulation model was further verified on predicting the spray rate of the boot shape spray in addition to the main and short sprays from Fig. 5.

Consider that the realization of driving voltage waveform with adjustable parameters and high-precision test of spray rate curve are not easy, the simulation model verified by experiments shown in Fig. 3 is employed to analyze the boot shape injection of the GMI fully.

### Adjustment of the “boot length”

**Figure 8:**
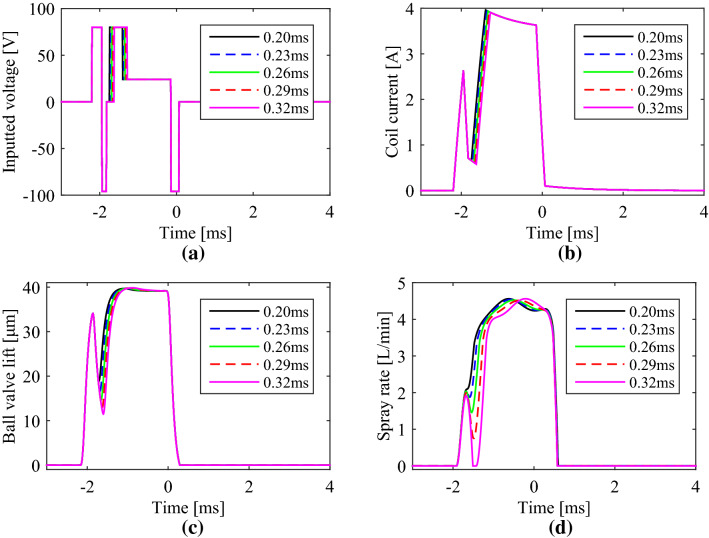
Boot shape injections with different dwell time from 0.20 ms to 0.32 ms: (**a**) driving voltage; (**b**) coil current; (**c**) ball valve lift; (**d**) spray rate.

Changing the dwell time between the pilot and main driving pulse width, the duration time of pilot spray, also represented as “boot length”, can be adjusted continuously. With the dwell time increased from 0.20 to 0.32 ms with the distance of 0.03 ms, simulating results were drawn in Fig. 8. The total time including the pilot segment and main segment was not changed by adjusting the hold time in the main injection segment correspondingly to take a fair contrast.

From simulation results, the coil current or ball valve lift reduced for longer time to lower values with the dwell time increased. Correspondingly, the “boot length” of the spray rate curve was elongated from 0.3 to 0.4 ms and the pilot spray quantity increased from 4.5 to 8.8 mm^3^ approximately. In addition, when the dwell time was 0.32 ms or higher, the pilot and main injection were completely parted. That is to say, through adjusting the dwell time of the driving voltage, the GMI can switch freely between a double injection and a boot shape injection.

### Adjustment of the “boot height”

**Figure 9:**
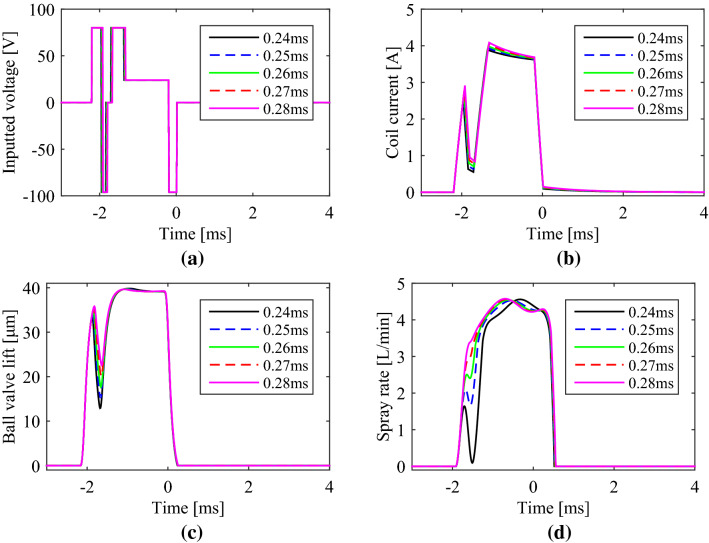
Boot shape injections with different open time in the pilot spray segment from 0.24 to 0.28 ms: (**a**) driving voltage; (**b**) coil current; (**c**) ball valve lift; (**d**) spray rate.

In addition to adjusting “boot length”, the spray rate amplitude of the pilot spray in the boot shape injection, represented as “boot height”, can also be adjusted continuously. With the open time of the pilot spray increased from 0.24  to 0.28 ms with the distance of 0.01 ms, simulation results from AMEsim model were shown in Fig. 9. The total time including the pilot segment and main segment was not changed by adjusting the hold time in the main spray segment correspondingly to take a fair contrast.

From simulated results, with the open time of the pilot spray increased from 0.24 to 0.28 ms, the mean value of “boot height” of the spray rate was promoted from 0.9 to 3.0 L/min approximately, and the pilot spray quantity increased from 5.8 to 11.0 mm^3^ approximately. Just as changing the dwell time, changing the open time in pilot injection segment was also qualified for various pilot spray quantities.

From above simulations for boot shape injections, designed GMI can realize a multi-injection or a boot-injection with appreciate driving voltage. Furthermore, the boot spray can adjust its “boot length” and “boot height” respectively through adjusting the dwell time and open time in the pilot segment of the driving voltage. It can be concluded that benefited from the accurately controlled output of the giant magnetostrictive actuator, the GMI can reach quite flexible spray rate to adapt to various injection requirements.

## Conclusions

This paper proposed a novel indirect acting GMI and appropriate driving voltage waveform for this type of injector. With the open-hold-fall and open-fall type of driving voltages, the injector was qualified for the main and pilot injection, respectively. Simulation model using AMEsim software and high pressure common rail test system for designed injector were established. From simulation and experimental results, designed GMI reached a boot shape injection when driven by a specific voltage wave and showed good performances as maximum injection rate of 4.5 L/min and minimum spray pulse width of 0.21 ms. In addition, As simulated spray rates corresponded to the experimental ones well, the model’s accuracy in predicting the injection characteristics of the GMI was verified. Then the boot shape injections with controllable parameters were studied using the simulation model. From simulation results, the injector realized adjustable boot shape injections with appreciate driving voltage. Through changing the interval time and open time, the length and height of the front end of the boot can be adjusted, respectively.

## Supplementary Information

Supplementary Information 1.

Supplementary Information 2.

## Data Availability

All simulation data generated or analysed during this study are included in this published article (and its Supplementary Information files 1 and S2).The other datasets generated during and/or analysed during the current study are available from the corresponding author on reasonable request.
