# Supplementary material for: Simulation studies on the boot shape injection of a giant magnetostrictive injector
Source: Sci Rep. 2021 Nov 26;11:22999. doi: 10.1038/s41598-021-02529-z (PMC8626452; doi:10.1038/s41598-021-02529-z)
Supplement: Supplementary file 1 — Supplementary Information 1. [file 41598_2021_2529_MOESM1_ESM.pdf]

In addition to directly using the source file, one can reconstruct the model in combination with the Sketch Mode model in the manuscript and the following parameter settings.

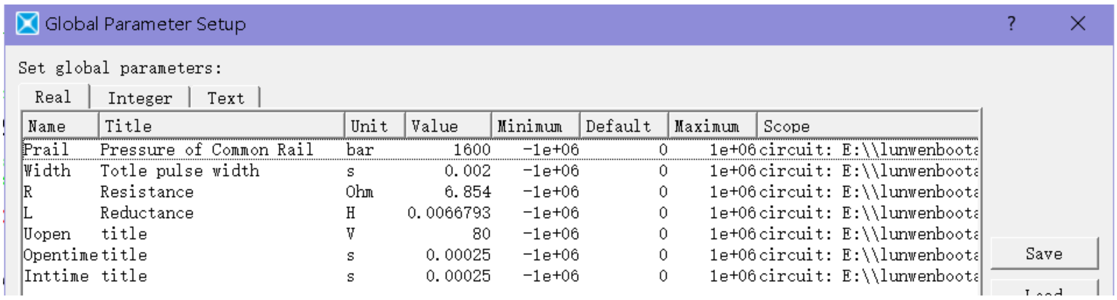

Figure S1. Global parameters

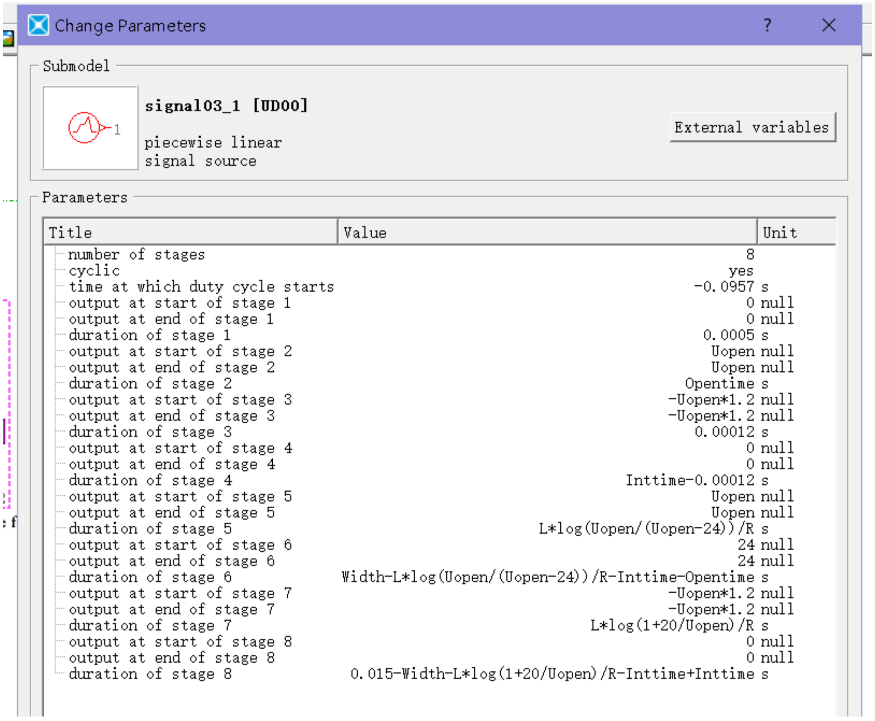

Figure S2. Driving voltage wave

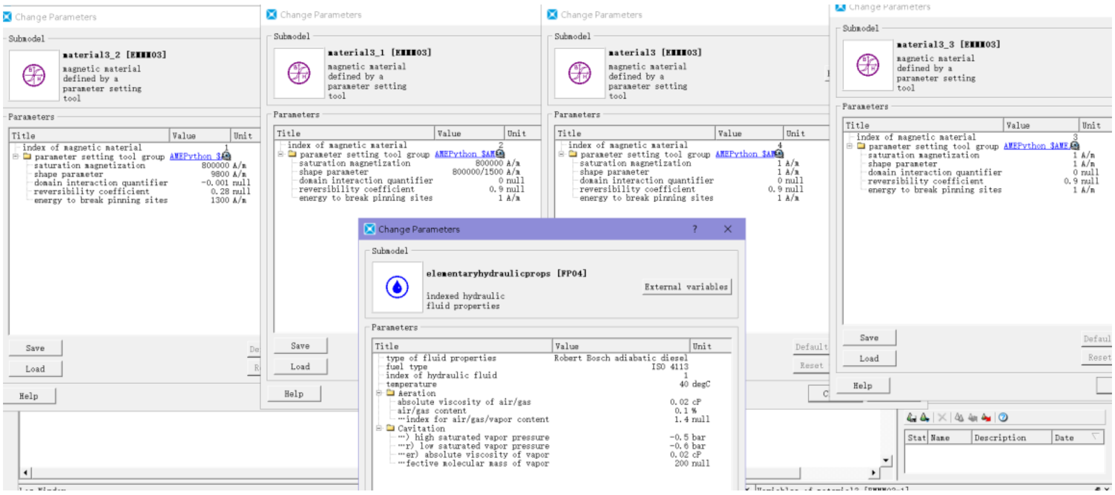

Figure S3. Material and fuel

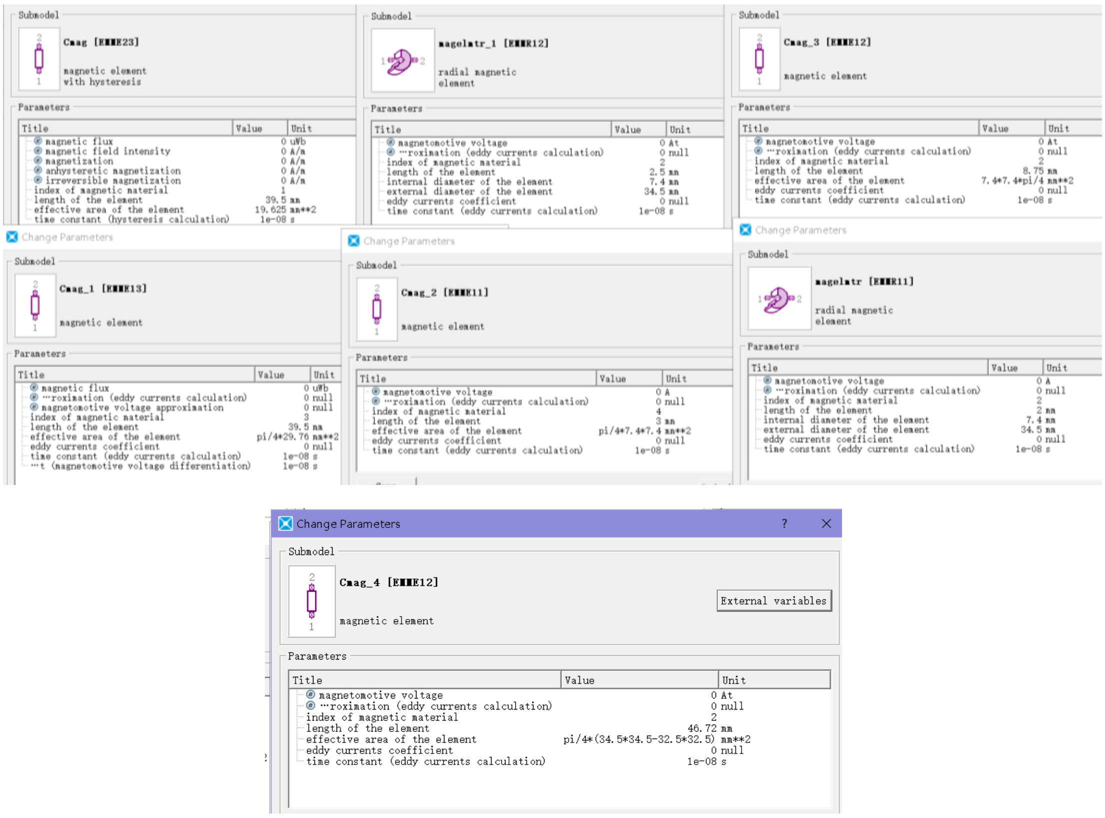

Figure S4. Magnetic circuit

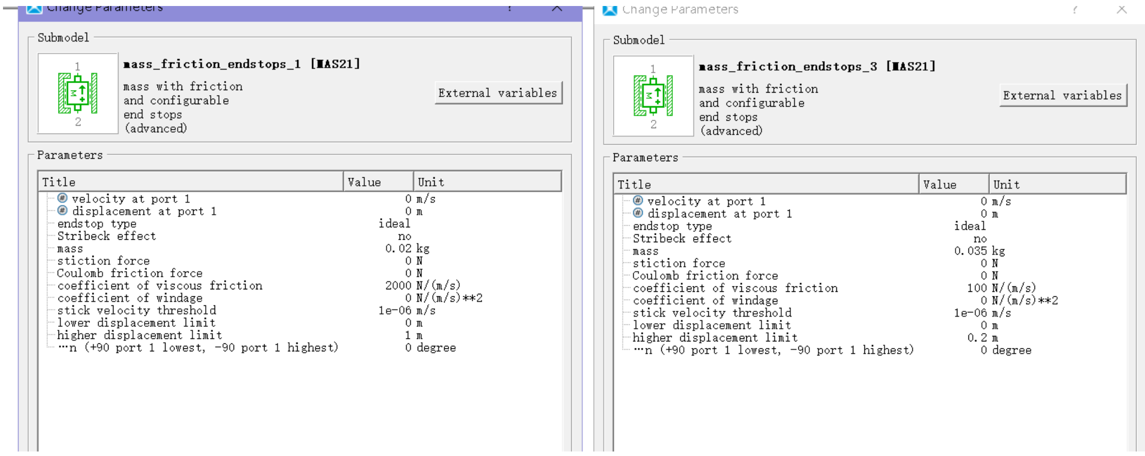

Figure S5. Masses

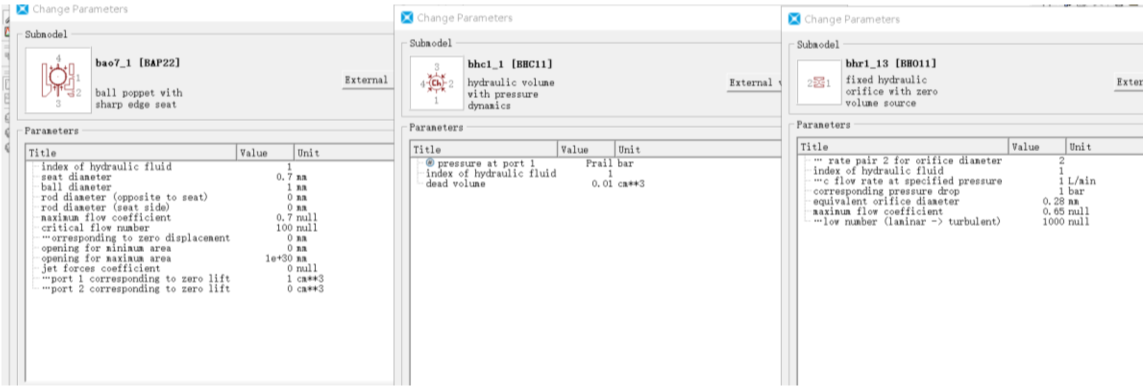

Figure S6. Ball valve

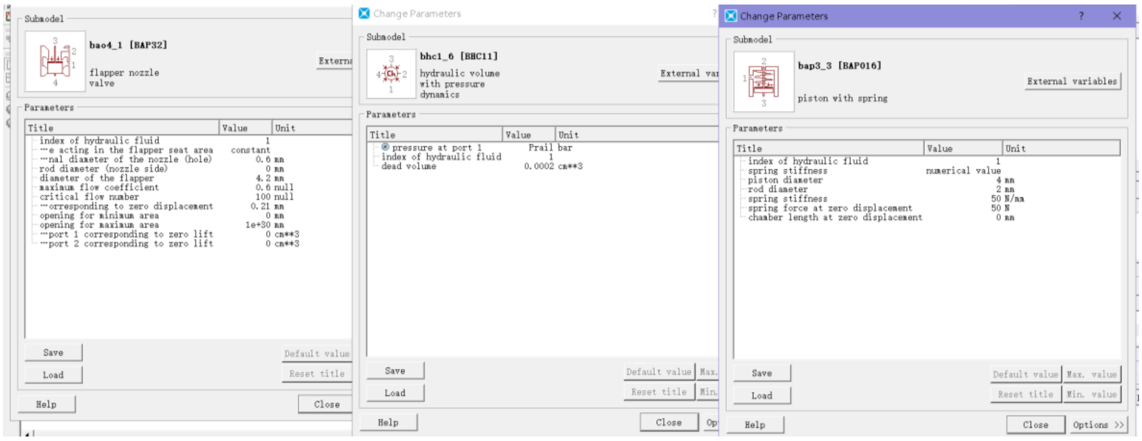

Figure S7. Control piston

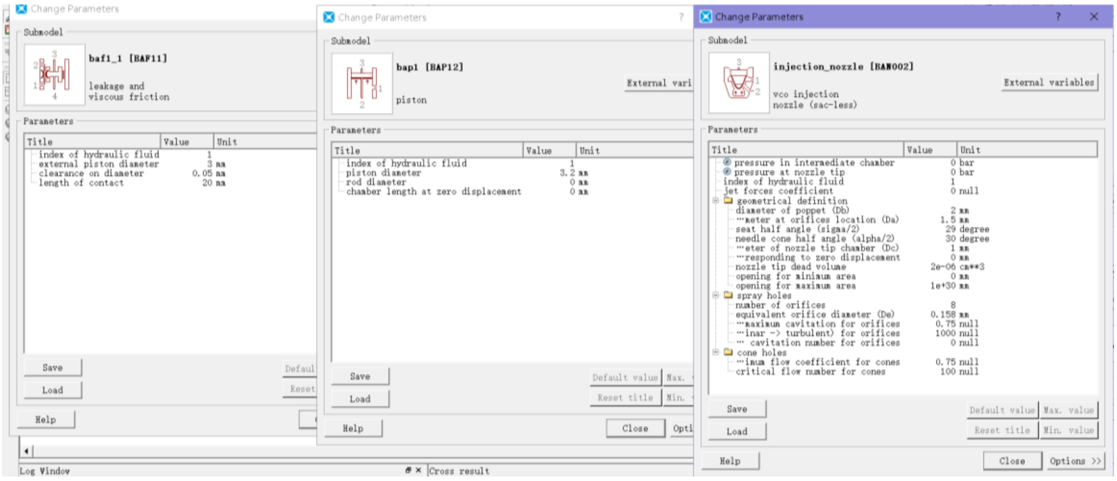

Figure S8. Needle valve
